# Multidrug resistant bacterial isolates causing nosocomial urinary tract infection in a tertiary care hospital, Nepal

**DOI:** 10.1186/2047-2994-4-S1-P222

**Published:** 2015-06-16

**Authors:** MK Sah

**Affiliations:** 1 Department of Clinical Microbiology, Kathmandu University, Kantipur Dental College Teaching Hospital & Research Center, Basundhara, Kathmandu, Nepal, Kathmandu, Nepal

## Introduction

Nosocomial infection is becoming a leading problem in medical practitioners now-a-days placing an extra burden on individual patients worldwide. Nosocomial urinary tract infection caused by multidrug resistant (MDR) pathogens is a major threat of the patients in developing country which are increasing numbers in Nepal.

## Objectives

The aim of this study was to determine the etiology of nosocomial urinary tract infection caused by multidrug resistant bacterial pathogens.

## Methods

A total of one hundred twenty two bacterial strains isolated from the patients diagnosed of nosocomial urinary tract infection were studied during 2011-2012 at Tribhuvan University Teaching Hospital (TUTH). Antibiotic sensitivity test was determined by modified Kirby Bauer Disc Diffusion method as described by Clinical and Laboratory Standards Institute (CLSI). Data were analysed by using SPSS version 17.0 software.

## Results

Nosocomial urinary tract infection was caused by *Escherichia coli* 51(41.8%) was found to be more predominant which was followed by *Acinetobacter calcoaceticus baumannii* (Acb) complex 19(15.6%), *Klebsiella pneumoniae* 11(9%) *Enterococcus* spp. 18(14.8%) and *Staphylococcus aureus* 11(9%). Of the total isolates, 74.6% was MDR which is much higher in *Klebsiella pneumoniae* 100% which was followed by *Escherichia coli* 90.1%.

## Conclusion

The emergence of MDR bacterial strains causing nosocomial urinary tract infection are increasing in number. The high prevalence of MDR has demanded the special attention to the management of such patients and prevention of dissemination of such strains into hospitals.

## Disclosure of interest

None declared.

